# Helping Health Services to Meet the Needs of Young People with Chronic Conditions: Towards a Developmental Model for Transition

**DOI:** 10.3390/healthcare5040077

**Published:** 2017-10-19

**Authors:** Albert Farre, Janet E. McDonagh

**Affiliations:** 1 Institute of Applied Health Research, University of Birmingham, Birmingham B15 2TT, UK; a.farre@bham.ac.uk; 2 Centre for Musculoskeletal Research, Division of Musculoskeletal and Dermatological Sciences, The University of Manchester, Manchester M13 9PT, UK; 3 NIHR Manchester Musculoskeletal Biomedical Research Centre, Central Manchester University Hospitals NHS Foundation Trust, Manchester M13 9WL, UK

**Keywords:** transition to adult care, adolescent development, psychosocial aspects, developmentally appropriate healthcare, adolescent medicine, adolescent health services, young adults, chronic illness, health care delivery

## Abstract

The transition to adult healthcare has been the subject of increased research and policy attention over many years. However, unmet needs of adolescent and young adults (AYAs) and their families continue to be documented, and universal implementation has yet to be realised. Therefore, it is pertinent to re-examine health transition in light of the principles of adolescent medicine from which it emerged, and consider this particular life transition in terms of a developmental milestone rather than a negotiation of structural boundaries between child and adult services. Health transitions are an integral part of AYA development and as such, occur alongside, and in connection with, a range of other important transitions that affect many other areas of life. In this paper, we discuss the interrelated nature of health transitions and AYA development; outline the underpinnings of a developmentally appropriate approach to transitional care; and consider the outcome measurement of such care based on existing evidence. A developmental approach has the potential to refocus transition on the fundamental principles of adolescent medicine, enabling health transition to be integrated along with other life transitions into routine AYA developmental assessments rather than being limited to the geographies of different healthcare settings and a potential health crisis.

## 1. Introduction

Transition in health literature is often considered synonymous with health transition, i.e., the preparation of young people and their families as they move from child to adult-centred services [1]. Transitional care not only prepares young people and their caregivers for the differences between child and adult services and how to negotiate them but also supports the development of health literacy and self-management skills. Such knowledge and skill development is vital at this life stage as responsibility shifts from parents to a shared responsibility and eventually, if they have capacity, to the young person themselves.

Health transitions have been discussed and debated for many years in the literature [2], resulting in national and international guidance [3,4]. In spite of this however, an evidence base has been slow to be established [1] and reports of unmet needs of young people and their families continue to be reported [5]. Barriers to implementation at professional and system levels also continue to be reported [6,7].

Perhaps it is therefore time to reconnect transition to the principles and practice of adolescent medicine from which it emerged [8] and consider health transition in terms of a developmental milestone rather than a negotiation of structural boundaries between child and adult services [9].

In this paper, we will (1) discuss the interrelated nature of health transitions and adolescent and young adult development; (2) outline the fundamental elements of a developmentally appropriate approach to transitional care; and (3) consider the outcome measurement of such care in light of existing evidence.

## 2. Health Transition as One of Multiple, Interrelated Transitions

Adolescents and young adults (AYAs) experience health related transitions as they undergo a range of developmental changes during adolescence and young adulthood, which in turn are associated with many other life transitions (Table 1).

Health transition is an integral part of development for all young people, with and without a long-term health condition. Young people frequently remind us that they are young people first and foremost—“it [transition] is not about arthritis, it is about living with it” [10]. Bearing this in mind, it is important to acknowledge the developmental context of health transition and consider how the different aspects of that development, including the transitions listed in Table 1, impact on health transitions and vice versa.

Unfortunately, key aspects of AYA development are not always adequately acknowledged in the health care of this age group. In a study of 290 young people, 227 of whom had a long-term health condition and a mean age of 17 years, key adolescent health-related issues were not universally addressed by staff [11]. There was also a significant discrepancy between reported discussion of psychosocial issues by professionals compared to young people, raising the issue of the efficacy of communication strategies being employed by staff [11]. Such routine psychosocial screening is core to transitional care [8] and yet not always covered by the current transition readiness tools [12].

Adopting a developmental model for transition addresses this by refocussing transitional care on a fundamental principle underpinning the practice of adolescent medicine, i.e., developmentally appropriate healthcare [13,14,15], and placing health transition within the wider context of AYA development—i.e., considering the impact of the biological, psychological, social and vocational aspects of AYA development on health transition as well as the impact of health transition on such development.

## 3. Developmentally Appropriate Transitional Care for Young People

The increasing knowledge on AYA development [16,17] and the inter-relationships of the different aspects of such development (e.g., impact of pubertal timing on social development [18,19]) offers unprecedented opportunities to develop and reshape health care services to better meet the biopsychosocial developmental needs of AYAs. However, it is important to develop a shared understanding in the clinical and organisational arena about what such adolescent-responsive health services look like in practice [15] including transitional care services.

Developmentally appropriate healthcare (DAH) for young people, a key principle underpinning the practice of adolescent medicine [13,20] is particularly well suited to serve this purpose. DAH is an approach to clinical work with adolescents and young adults, which conveys the dynamic nature of AYA development (rather than chronological age) as a defining characteristic of health services, and offers room to achieve consistency in clinical practice regardless of whether adolescent medicine is recognised as a distinct specialty or not in a particular context [21].

Although a scoping review concluded that there is still the need to further clarify and operationalise the definition of DAH to enable a more consistent use of the term in the literature and future research [14], a subsequent ethnographic study of health professionals and managers responsible for delivering such care identified five conceptual dimensions which potentially provide a framework for such care provision [15]:

1. *Biopsychosocial development and holistic care*. Key developmental milestones of AYAs at biological, psychological, social and vocational levels (across early, mid and late adolescence and emerging adulthood) inform clinical work by either (1) adopting a holistic clinical approach that looks beyond the physical aspect of one’s condition and integrates the biological psychological, social and vocational aspects of development; or (2) incorporating additional care components to otherwise standard paediatric/adult care. Family or trusted others are included as key stakeholders and/or active participants of healthcare provision for AYAs [15].
2. *Acknowledgement of AYA as a distinct group*. Developmental needs of AYAs inform distinct ways of interacting and communicating with them, in terms of (1) what, when and how information is given to and gathered from them; and (2) official communication materials and means (such as appointment letters or text message reminders). Specific/tailored services, spaces and pathways are provided. The distinct needs of the service (e.g., need for longer appointments) and those of the health professionals looking after AYAs (e.g., training in adolescent health) are acknowledged and addressed [15].
3. *Adjustment of care as the young person develops*. The starting point of DAH is a developmental assessment, covering all areas of AYA development and actively involving parents/carers and/or trusted others. Routine follow-up developmental assessments are then undertaken and used to inform the tailoring of particular aspects of clinical work and service delivery as the young person grows up [15].
4. *Empowerment of the young person by embedding health education and health promotion*. AYAs are routinely provided with (1) informal education on self-management skills and (2) health promotion information and lifestyle behaviour change advice relevant to each stage of development to enable AYAs to make informed choices. Health education and health promotion delivered in the context of DAH services is informed by, but not limited to, a health transition agenda. The approach to health education and health promotion delivered in the context of DAH is based on promoting active engagement and autonomy-enabling practices without creating relations of dependency with the service/clinical team [15].
5. *Interdisciplinary and interorganisational work.* Effective multidisciplinary work both within and across services, teams, specialities and organisations [15].

In Table 2, we provide some examples as to how these dimensions are translated into practice.

**Figure 1:**
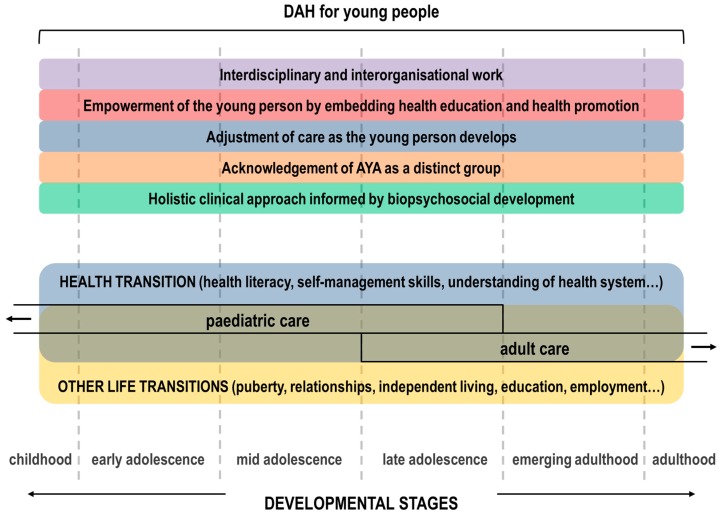
Schematic representation of the relationship of the five conceptual dimensions of DAH for young people to health transition and adolescent development.

Following the description of the five conceptual dimensions of DAH for young people outlined above and the examples listed in Table 2, it becomes clear that the provision of DAH in paediatric and adult settings encompasses transitional care by definition. Health transitions are an integral part of AYA development that will be integrated along with other life transitions into routine developmental assessments (Figure 1).

Alongside these considerations, it is equally important to consider the perspectives of AYAs themselves. Listening to the views of young people and their caregivers is essential to the development of meaningful services and this is reflected in the literature addressing the principles of youth-friendly health care (YFHC) [24]. As with transitional care, national and international guidance has existed in the literature detailing the core indicators of YFHC [24,25,26], which can be used to ensure that transitional care services are also youth-friendly (Table 3).

Thus, the different emphases of DAH and YFHS (i.e., the emphasis on DAH as compared to YFHC is shifted from the service and centres on the development of the young person) can be combined to provide health services that are youth-friendly and developmentally appropriate, i.e., services that are meaningful and consistent from both the AYA and provider perspectives. This echoes the WHO call to move from adolescent-friendly service delivery into services that can respond to the priority health and development needs of adolescents [27]. Therefore, it is paramount to ensure that transitional care services are adolescent-friendly in the first place and that they continue to be adolescent-friendly as they transform into developmentally appropriate services.

If one adopts the concept of developmentally appropriate transitional care services, when a transition intervention is proven to be ineffective, a new range of potential explanations can be considered:

- Was the intervention concerned with changing the approach to clinical work based on key developmental milestones of AYAs from early adolescence through to emerging adulthood?
- Were the family or AYA’s trusted others included in service provision?
- Was the individual and service level communication strategy tailored to developmental needs?
- Were AYA-specific/tailored services, spaces and pathways provided or engaged with as part of the intervention?
- Were the needs of staff delivering the intervention dealt with (adolescent health training, availability of resources) as part of the intervention?
- Were routine and follow-up biopsychosocial developmental assessments (and tools) built into the intervention and used to inform dynamic changes in clinical work and service delivery as AYAs develop?
- Was the intervention concerned with supporting effective multidisciplinary work both within and across services, teams, specialities and organisations?

These are all questions mainly related to intervention delivery, the ‘whys’ and ‘hows’ of a given set of outcomes, and can be explored through process evaluation designs.

## 4. Outcome Indicators of Transition

The ultimate aim of both transitional care and/or DAH for young people with long-term conditions is improvement in their health-related outcomes. There is increasing interest in the selection of outcome indicators of transition.

In a recent Delphi process involving professionals from outpatient, community-based and primary care settings, 10 outcomes were identified, with the majority being health service related, except for ‘optimal quality of life’ and ‘a social network’, and none including vocational or psychological outcomes [28]. Similarly, the four randomised controlled trials identified in the recent Cochrane review [1] primarily employed health service related measures, with the exception of the PedsQol, as did Sharma et al. [29]. The proposed indicators from these three contributions are summarised in Table 4, alongside the ones proposed in current NICE guidelines.

The term transition readiness is increasingly prevalent in the literature although still in its infancy [12]. At times, it is unclear as to whether this is truly ‘transition readiness’ or simply ‘transfer readiness’. Furthermore, it is unclear as to whether this readiness is being considered in the context of AYA development. What is the exact nature of this readiness—physical, psychological and/or social readiness? Few measures consider educational and vocational development, which is concerning in view of the finding that poor health in adolescence is associated with poorer education and poorer employment outcomes in adulthood [30]. Although young people with long-term conditions have similar social success to controls, they have lower rates of mastering vocational milestones [31,32].

Many of the readiness tools under development are self-reported measures. This raises the question as to how mastery of the skills is assessed. Fredericks et al. [33] reported the interesting finding that although young people aged 16–20 years with liver transplants perceived greater self-management on a transition readiness survey, they had a higher risk for medication nonadherence.

In addition to consideration of assessment of mastery of skills which are practiced beyond the clinic setting (such as adherence to medication or ability to order medication refills), attention is also required as to whether the setting in which these skills are being practiced is a place where such skills are promoted (including resilience promoting personnel). This includes health care settings as well as the home and school setting. A young person may be ‘transition-ready’ but may be unable to practice their skills due to the lack of promotion of positive youth development in a particular health care setting. For example, if young people are not routinely offered the opportunity to ask their own questions with care givers present (and/or be seen independently of their caregivers), they will be unable to practice asking questions about sensitive topics. Likewise, if caregivers continue to take full responsibility for the administration of daily medication in the absence of any limited capacity of the young person to learn to do so, the young person may never acquire the skills to take responsibility themselves. This is particularly pertinent in view of the reports that self-efficacy and resilience are useful predictors of health transition readiness [34].

Several studies have reported a significant and positive relationship between age and skill acquisition [35,36,37,38,39] with age predicting mastery of skills over time [35]. Previous studies also found that few AYAs achieve mastery of health care transition skills by age 18, especially in the domains of ‘disease self-management’ and ‘understanding of health insurance’ [36,37,38] emphasising the need to acknowledge that health transition does not end with the event of transfer but extends into young adulthood and hence adult services.

It is important to remember that all young people need skill development in health management irrespective of whether they have a long-term condition or not. Eaton et al. [40] reported that AYAs with medical conditions (mean age 19 years) reported significantly higher levels of health transition readiness and self-involvement in completing medical tasks, and lower levels of parental involvement in completing medical tasks, than healthy peers. Equally, it is important to note that as with all developmental milestones, health transition readiness may regress. More prospective research in the area of health transition readiness trajectories is awaited with interest.

Another area of outcome research is consideration of the different perspectives involved, particularly those of AYAs themselves and their parents. In the Fair et al. study [28], there was a low representation of AYAs and their family members beyond stage 1 of the Delphi process, as noted by the authors. A more recent study by Pierce et al. [41] involved 10 young adults and 9 parents, but the study focused on young adults who had recently transferred out of paediatric care. AYAs from a range of ages and backgrounds may have an equally wide range of opinions as to the choice of outcome measures, i.e., what is important, in their opinion at this particular time in their lives, for health professionals to measure.

This relates to another important question: when is the right time to measure? One could argue that readiness is more a process rather than an outcome measure and that there are different stages of readiness just as there are different stages of adolescent and young adult development. Reflecting this, the NICE guidance for health and social care service transition in England emphasises that measurement requires an adequate duration to capture relevant condition-specific and quality of life outcomes [4].

Failure to attend appointments is a familiar outcome measure and is often considered as a negative outcome. However the reason given by young people for this nonattendance may be work and school conflicts [42] or a fear of being judged for “poor control” [43] and therefore more a reflection on the accessibility and developmentally appropriateness of the service rather than a failure of health transition readiness of the young person themselves.

Sharma et al. [29] also included professional and system-related outcome measures, which aligns with the dimensions of DAH detailed above, acknowledging the role of the team and organisation in transitional care (Table 5).

Outcomes, therefore, need to reflect the different aspects of DAH, i.e., medical (health and illness), psychological, social, educational/vocational and acknowledge the different levels, i.e., young person and family, care team, organisation (including setting where care is delivered) and environment in terms of regulatory and policy framework.

## 5. Conclusions

Transition to adult healthcare has been the subject of increased research and policy attention over many years, and yet unmet needs of young people and their families continue to be documented and universal implementation has yet to be realised. Health transitions are an integral part of AYA development and as such, occur alongside, and in connection with, a range of other important transitions that affect many other areas of life.

Adopting a developmental model for health transition in both clinical practice and research refocuses transitional care on a fundamental principle underpinning the practice of adolescent medicine, i.e., developmentally appropriate healthcare [13,14,15], and places health transition within the wider context of AYA development. The impact of the biological, psychological, social and vocational aspects of AYA development on health transition is therefore routinely considered as well as the impact of health transition on such development. In doing so, health transition is integrated along with other life transitions into routine AYA developmental assessment and ceases being limited to the negotiation of structural boundaries and a potential health crisis.

## Figures and Tables

**Table 1:** Some key transitions associated with developmental stages of adolescence and young adulthood.

Developmental Stage	Transitions
Early Adolescence 10–13 years	Biological (e.g., early puberty)
	Psychological (e.g., concrete thinking to early moral concepts)
	Social (e.g., emotional separation from parents)
	Health (e.g., early self-management)
	Educational (e.g., primary to secondary school)
Mid Adolescence 14–16 years	Biological (e.g., mid-late puberty)
	Psychological (e.g., early abstract thinking)
	Social (e.g., strong peer identification)
	Health (e.g., increased self-management)
	Educational/Vocational (e.g., school to college)
Late adolescence 17–19 years	Biological (e.g., completion of puberty)
	Psychological (e.g., complex abstract thinking)
	Social (e.g., independent living and travel)
	Health (e.g., paediatric to adult healthcare)
	Educational/Vocational (e.g., college to further education or training)
Emerging Adulthood 20–24 years	Biological (e.g., completion of brain development)
	Psychological (e.g., exploration of self-identity)
	Social (e.g., independent living and financial independence)
	Health (e.g., autonomous self-management)
	Educational/Vocational (e.g., further education/training to employment)

**Table 2:** Some examples of how the five conceptual dimensions of developmentally appropriate healthcare (DAH) for young people are translated into practice.

Conceptual Dimensions of DAH for Young People [15]	Examples of How These Dimensions Are Translated into Practice
biopsychosocial development and holistic care	Routine biopsychosocial developmental assessment and approach to the young person adjusted accordingly Use of psychosocial screening tools such as the HEEADSSS (Home environment, Education/employment, Eating, Activities, Drugs, Sexuality, Suicidal ideation, and Safety) psychosocial interview [22,23] as a routine component of clinical consultations with adolescent and young adults (AYAs)
acknowledgement of young people as a distinct group	Acknowledgement of age and developmental stage-specific issues and how these may change during adolescence and young adulthood: Confidentiality and rights Privacy issues Peer support Accessible services (e.g., after school/college) Dedicated clinics/space Longer appointment times to enable time to see young person independently of parents Information and resources
adjustment of care as the young person develops	Flexibility in approach and acknowledgement of regression during active phases in relapsing conditions: Change in communication style with respect to cognitive development Impact of physical growth and pubertal stage on condition and therapy
empowerment of the young person by embedding health education and health promotion	Support for the young person and parent as they move from shared to self-management of health (within their individual capacity). This includes knowledge and skill development in both health and disease management as well as health care utilisation skills Information and resources
interdisciplinary and interorganisational work	Effective team working Staff training in adolescent health AYA responsive staff and services AYA-specific issues addressed in policies and guidelines; Support of key interfaces with education and vocational agencies, social care, youth work reflecting holistic approach to care Coordination/continuity/consistency across roles, professionals and shifts Coordination/continuity/consistency throughout a patient’s journey through the health care system

**Table 3:** Summary domains of adolescent-friendly care, with examples of relevant indicators [24].

Domains of youth-friendly health care (YFHC)	Examples of Relevant Indicators
1. Accessibility of health care	Location, affordability
2. Staff attitude	Respectful, supportive, honest, trustworthy, friendly
3. Communication	Clarity and provision of information, active listening, tone of communication
4. Medical competency	Technical skills (procedures)
5. Guideline-driven care	Confidentiality, autonomy, transition to adult health care services, comprehensive care
6. Age-appropriate environment	Flexibility of appointment times, separate physical space, teen-oriented health information, cleanliness, waiting time, continuity of care, privacy
7. Involvement in own health care	Understanding of one’s medical condition and treatment; acquisition of self-management skills
8. Health outcomes	Pain management, quality of life

**Table 4:** Summary of the proposed outcome indicators of transition in the current literature.

Outcome	Campbell et al. 2016 [1]	Fair et al. 2015 [28]	Sharma et al. 2014 [29]	NICE 2016 [4]
Transition readiness, self efficacy	Transition readiness (TRAQ); Patient Activation Measure; Community Life Skills (CLSS); Self-care practice	Self-management; Adherence to medication/treatment	Transition readiness	Transition readiness; Self-efficacy (YP’s ability to undertake the activities they want to, as independently as possible)
Disease-specifc status	HbA1C	−	Condition-specific outcomes	Condition-specific outcomes
Well being	Personal Adjustment and role skills (PARS III); Peds Qol	Achieving optimal Quality of life (QoL)	QoL	Qol (health and social care indicators)
Knowledge of disease and treatment	MyHeart	Disease knowledge; Medication knowledge	−	−
Knowledge of transition	−	−	Transition knowledge	−
Transfer from paediatric to adult services	% young people successfully transferred to adult services	−	Gaps in medical care	Continuity of care (loss of contacts with services, lack of appropriate referral, satisfaction, interagency communication, clinical outcomes)
Healthcare utilisation	Patient initiated health care communication; Hospitalisation	Attending medical appointments; Having a medical home; Avoiding unnecessary hospitalisations	Health care utilisation; Gaps in medical care	Health and social care resource utilisation
Understanding health insurance	−	understanding health insurance options	Loss of health insurance; Health coverage issues	−
Having a social network	−	Having a social network of friends	−	−

**Table 5:** Sharma et al.’s [29] outcome indicators for the role of the team and organisation in health transition.

Stage of Health Transition	Health Care Professional	Health System
Preparation	Use of transition care plans	Development of transition policy
	Assessment of transition readiness	
	Young person/family education and counselling	Care coordination
Transfer of care	Preparation of patient summary	Use of patient summary
	Communication	Communication between paediatric-adult systems
Post transfer	Intake policy for transferring patients	Quality of intake to adult care
		Care coordination
		Financial costs and savings
